# Anastrozole-induced autoimmune hepatitis: a rare case report and literature review

**DOI:** 10.1093/omcr/omaf062

**Published:** 2025-05-28

**Authors:** Shahab Haghollahi, Rebecca M Wood, Abdol Aziz Ould Ismail, Bing Ren, Muhammad Z Afzal

**Affiliations:** Department of Medicine, Dartmouth-Hitchcock Medical Center at Dartmouth Geisel School of Medicine, Hanover, NH 03756, United States; Department of Medicine, Dartmouth-Hitchcock Medical Center at Dartmouth Geisel School of Medicine, Hanover, NH 03756, United States; Department of Pathology and Laboratory Medicine, Dartmouth-Hitchcock Medical Center at Dartmouth Geisel School of Medicine, Hanover, NH 03756, United States; Department of Pathology and Laboratory Medicine, Dartmouth-Hitchcock Medical Center at Dartmouth Geisel School of Medicine, Hanover, NH 03756, United States; Department of Hematology and Oncology, Dartmouth Hitchcock Cancer Center at Dartmouth Geisel School of Medicine, Hanover, NH 03756, United States

**Keywords:** Anastrozole, autoimmune hepatitis, aromatase inhibitors, breast cancer, liver injury

## Abstract

Background: Anastrozole, an aromatase inhibitor (AI), has been used extensively for the treatment of estrogen receptor-positive breast cancer. Autoimmune hepatitis (AIH) is an extremely rare but serious complication of anastrozole treatment. Case description: We present the case of an 81-year-old female who presented with significantly elevated liver function test (LFTs) results 8 months after the initiation of anastrozole for early-stage breast cancer. Serological and liver biopsy findings were consistent with AIH. Discontinuation of anastrozole, along with a short course of steroids, resulted in rapid clinical improvement and normalization of both LFTs and autoantibodies. Conclusion: Clinicians should be aware of drug-induced AIH as a rare but life-threatening complication of anastrozole and potentially other aromatase inhibitors.

## Introduction

Breast cancer is the second most common cancer among women in the United States and causes up to 42 000 deaths annually [1]. Anastrozole, a nonsteroidal aromatase inhibitor (AI), is a commonly used medication for hormone receptor-positive breast cancer in postmenopausal women. It reduces plasma estrogen levels by blocking the peripheral aromatization of androgens to estrogen. The most common side effects of anastrozole are osteoporosis, arthralgia, myalgia and hypercholesterolemia [2]. Abnormal liver function tests (LFTs) have been reported in approximately 2%–4% of patients treated with anastrozole, although these elevations are usually mild, asymptomatic, and self-limited [3]. Only three cases of autoimmune hepatitis (AIH) have been previously reported [4–6]. Herein, we report a case of AIH in a patient who received anastrozole.

## Case report

An 81-year-old female was found to have asymmetry in the outer left breast on a screening mammogram in February 2023. Biopsy revealed low-grade stage IA invasive ductal carcinoma. The cancer was estrogen and progesterone receptor-positive, and human epidermal growth factor 2/neu-2 negative. She had no history of liver disease, autoimmune conditions, or significant alcohol intake. Her medications included aspirin, lisinopril, sertraline, desipramine, acetaminophen, and multivitamin. She was not taking any herbal supplements.

The patient underwent left partial mastectomy in May 2023. The pathological stage was pT1cNxMx. She received accelerated partial breast irradiation (30 Gy in 5 fractions) and was started on a generic form of adjuvant anastrozole 1 mg in July 2023.

In March 2024, the patient presented to the clinic with 1 week history of dark urine, decreased appetite, fatigue, and generalized weakness. The patient denied excessive alcohol intake or herbal supplements. On physical examination, she was anicteric. The abdominal examination was benign and she had no peripheral edema.

**Figure 1:**
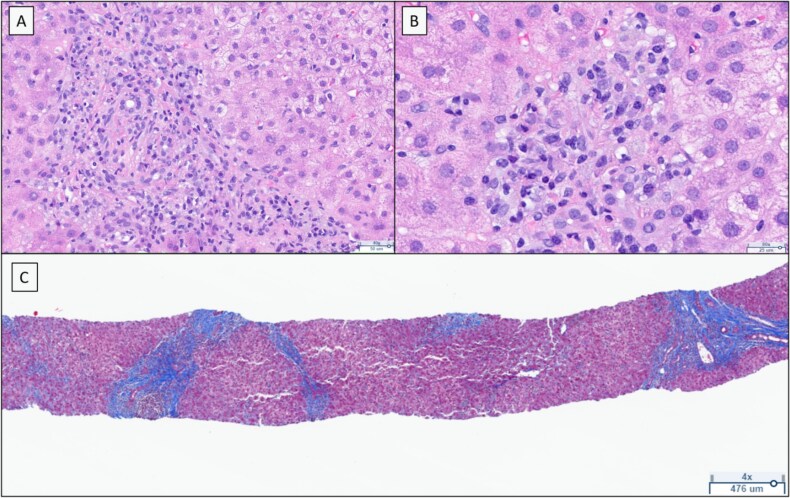
The liver parenchyma demonstrates a pronounced portal and lobular lymphoplasmacytic infiltrate, accompanied by focal interface hepatitis, mild ductal proliferation in portal area (A) and lobular confluent necrosis (B). The trichrome stain reveals prominent portal fibrosis, pericellular fibrosis, and focal bridging fibrosis (C).

Laboratory results showed significantly elevated liver function tests (LFTs): ALT 1172 IU/l, AST 988 IU/l, alkaline phosphatase 474 IU/l, total bilirubin 1.4 mg/dl. Synthetic liver function was preserved (Table. 1). Serum acetaminophen levels were undetectable. LFTs 4 months prior were normal: ALT 22 IU/l, AST 18 IU/l, alkaline phosphatase 82 IU/l, total bilirubin 0.2 mg/dl. Owing to concerns regarding possible drug-induced liver injury, anastrozole was discontinued. She was hospitalized and received intravenous N-acetylcysteine (NAC) per protocol (150 mg/kg, followed by 100 mg/kg over 2 days). Further laboratory testing showed a positive anti-smooth muscle antibody (titer 1:640) and an elevated IgG level of 1854 mg/dl. Anti-nuclear antibody (ANA), anti-double stranded DNA antibody (dsDNA) and mitochondrial antibodies were negative. Serum iron and alpha-1 antitrypsin levels were within normal limits. The viral hepatitis screening result was negative (Table. 2). A liver ultrasound with Doppler study was normal, and there was no evidence of portal vein thrombosis. She was discharged from the hospital two days after admission and underwent percutaneous liver biopsy, which showed lobular changes with brisk portal and lobular lymphoplasmacytic cellular infiltrates associated with interface hepatitis, confluent necrosis, and deposits of epithelioid histiocytes (Fig. 1).

**Table 1:** The patient’s complete blood count, renal function, and liver function on presentation.

Test	Result	Test	Result
Hemoglobin	13.3 g/dl (normal 11.7–15.5)	ALT	1172 U/l (normal 0–30)
White cell count	6.9 × 10^3^/μl (normal 4.0–9.5)	AST	988 U/l (normal 0–30)
Platelets	261 × 10^3^/μl (normal 145–357)	ALP	474 U/l (normal 35–105)
Serum creatinine	0.75 mg/dl (normal 0.70–1.20)	Total bilirubin	1.4 mg/dl (normal 0.2–1.3)
INR	1.0	Direct bilirubin	0.7 mg/dl (normal 0.0–0.3)
Albumin	3.6 g/dl (normal 3.2–5.2)		

INR, international normalized ratio; ALT, alanine transaminase; AST aspartate transaminase; ALP, alkaline phosphatase

**Table 2:** Additional diagnostic liver workup.

Test	Result
Anti-nuclear antibody	Negative
Anti-smooth muscle antibody	Positive (titer 1:640)
Mitochondrial antibody	Negative (< 0.1 U)
Immunoglobulin G	1975 mg/dl (normal 700–1600 mg/dl)
Serum acetaminophen level	< 5 mg/l
A1AT	200 mg/dl (normal 90–200)
HAV IgM antibody	Negative
HBS antigen	Negative
HCV antibody	Negative
HIV antibody	Negative
EBV IgM antibody	Negative
CMV IgM antibody	Negative
HSV-1 IgG antibody	Negative
HSV-2 IgG antibody	Negative

A1AT, alpha-1 antitrypsin; HAV, hepatitis A virus, HBS, hepatitis B surface antigen; HCV, hepatitis C virus; EBV, Epstein–Barr virus; CMV, cytomegalovirus; HSV, herpes simplex virus

**Figure 2:**
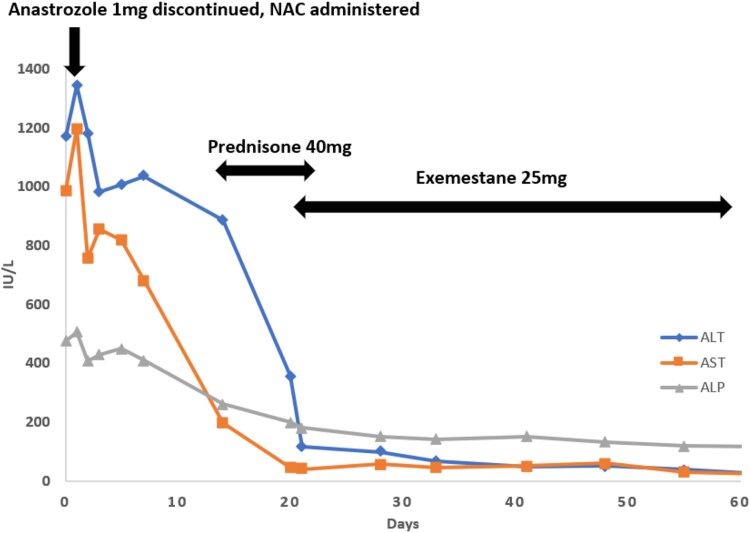
Timeline and trends of liver function tests after discontinuation of anastrozole, initiation of steroid, and transition to exemestane. ALT, alanine transaminase; AST, aspartate transaminase; ALP, alkaline phosphatase; NAC, n-acetylcysteine.

Based on the simplified diagnostic criteria for AIH, the patient’s total score was 7, which is highly suggestive of AIH [7] (Table. 3). After discontinuation of anastrozole and a 7-day course of prednisone 40 mg, the patient clinically improved, and LFTs showed a significant downward trend (Fig. 2). Given her rapid improvement and patient reservations regarding long-term steroid therapy, prednisone was discontinued. She was started on exemestane, a steroidal AI that she tolerated without issue. Her LFTs were completely normalized in June 2024, repeat anti-smooth muscle antibody test results were negative, and IgG levels were within normal limits.

**Table 3:** Simplified diagnostic criteria for autoimmune hepatitis.

Variable	Cut-off	Points
Antibodies	ANA or SMA ≥ 1:40	1
IgG	> 1.10 times upper normal limit	2
Liver histology	Typical AIH	2
Absence of viral hepatitis	Yes	2
Total score	≥ 6: probable AIH	7
	≥7: definite AIH	

Reproduced from Hennes *et al* [7]

## Discussion

Postmenopausal women with early-stage hormone receptor-positive breast cancer often receive adjuvant AI to decrease the risk of locoregional and distant recurrence [8]. LFT abnormalities are relatively rare (2%–4%) and are usually mild and self-limited. Hepatic steatosis has also been reported in approximately 9% of the cases [3]. To our knowledge, only three cases of anastrozole- and one case of letrozole-induced AIH have been reported [4–6, 9].

The onset of AIH symptoms and LFT elevation after anastrozole initiation in our patient was 8 months, compared to 4–9 months in previous cases. All three anastrozole-related AIH cases were positive for ANA antibodies, and only one case was positive for ASMA antibodies [4]. In contrast, in our patient, the ANA antibody was negative, but the ASMA antibody was positive, with a high titer (1:640). Interestingly, Inno *et al*. [5] reported that ANA titers declined after anastrozole withdrawal. In all three cases, clinical outcomes were favorable with normalization of LFTs after discontinuation of anastrozole, although the subsequent immunomodulatory strategies differed. These cases are summarized in Table 4.

**Table 4:** Clinical characteristics of previously reported cases of aromatase inhibitor-induced AIH.

Study	Sex/age	Latency	Aromatase inhibitor (dose)	ANA (titer if positive)	ASMA (titer if positive)	IgG (upper limit of normal)	Biopsy	Treatment	Outcome
Islam *et al.* [5]	66F	6 months	Anastrozole (N/A)	+ve (1:160)	+ve (1:80)	2110 mg/dL (1650 mg/dL)	Heavy portal tract mixed inflammatory cell infiltrate, of plasma cells and lymphocytes. Scattered eosinophils and neutrophils, interface hepatitis.	Anastrozole discontinuation, NAC, vit K, ursodeoxycholic acid	Relapsed 12 months later, resolved with prednisolone 40mg
Inno *et al.* [6]	70F	4 months	Anastrozole (1 mg/day)	+ve (1:80)	-ve	N/A	Moderate-severe fibrosis, moderate inflammatory activity	Anastrozole discontinuation	Resolved, switched to tamoxifen.
Klapko *et al.* [7]	71F	9 months	Anastrozole (1mg/day)	+ve (1:1280)	-ve	Normal	Chronic hepatitis with an increased number of plasma cells at the prtal-lobular interface and focal rosetting characteristic of AIH	Anastrozole discontinuation, prednisone taper (3 months), azathioprine	Resolved but developed cutaneous lupus erythematosus
Gharia *et al.* [11]	70F	3 months	Letrozole (N/A)	+ve (1:160)	36 units (ULN 19 units)	N/A	Portal tract and lobular inflammation with predominantly neutrophils, in addition to lymphocytes, eosinophils and fewer plasma cells.	Letrozole discontinuation.	Resolved
Current case	81F	8 months	Anastrozole 1 mg/day	-ve	+ve (1:640)	1854 mg/dL (1600 mg/dL)	Brisk portal and lobular lymphoplasma cellular infiltrate with interface hepatitis and confluent necrosis	Anastrozole discontinuation, NAC, prednisone 40mg (1 week)	Resolved, switched to exemestane

In our patient, the onset of liver injury after initiation of anastrozole, the pattern of LFT elevation, positivity for ASMA, liver biopsy findings, rapid improvement in LFTs on cessation of anastrozole, and a short course of steroids all seem to point to anastrozole-induced AIH. Normalization of IgG levels and negative ASMA two months after stopping anastrozole further support this diagnosis. Other causes of autoimmune liver disease were ruled out by serology and biopsy. Our patient was older than those in the other three cases described. This is an atypical age group for AIH, as this disease has a bimodal age pattern in females, one peak in the late teens, and one around menopause [8]. The serologic pattern observed in our patient (ANA-negative/ASMA-positive) was also different from the other cases that were all ANA-positive. There are no reported significant differences in clinical or histopathological findings or disease outcomes in ANA-positive and ANA-negative patients; however, ANA-positive patients tend to have higher IgG levels and higher rates of other autoimmune conditions [10]. The mechanism by which anastrozole induces AIH is not well understood. It has been hypothesized that anastrozole can affect the immune system by impairing self-tolerance, thereby triggering AIH [4].

In summary, AIH is an extremely rare complication of anastrozole-based therapy. When patients with anastrozole present with elevated LFTs, AIH should be suspected once other common causes of liver injury have been excluded. Immunological assessment with ANA, ASMA, and quantitative immunoglobulins can provide early clues. When there is diagnostic uncertainty, liver biopsy should be performed to help establish the diagnosis. Prompt discontinuation of anastrozole and, in severe cases, immunomodulatory therapy can rapidly reverse the course. Further studies are needed to determine the exact mechanism by which anastrozole induces AIH.
